# Oxidation Stability of Pig Liver Pâté with Increasing Levels of Natural Antioxidants (Grape and Tea)

**DOI:** 10.3390/antiox4010102

**Published:** 2015-01-27

**Authors:** Mirian Pateiro, José M. Lorenzo, José A. Vázquez, Daniel Franco

**Affiliations:** 1 Centro Tecnológico de la Carne de Galicia, Parque Tecnológico de Galicia, San Cibrao das Viñas, 32900 Ourense, Spain; E-Mails: mirianpateiro@ceteca.net (M.P.); jmlorenzo@ceteca.net (J.M.L.); 2 Grupo de Reciclado y Valorización de Residuos (REVAL), Instituto de Investigaciones Marinas (IIM-CSIC), Eduardo Cabello, 6, 36208 Vigo, Spain; E-Mail: jvazquez@iim.csic.es

**Keywords:** antioxidant level, storage time, oxidative stability, volatile compounds

## Abstract

The present study investigated the effect of the addition of increasing levels of the natural antioxidants tea (TEA) and grape seed extracts (GRA) on the physiochemical and oxidative stability of refrigerated stored pig pâtés. In addition, a synthetic antioxidant and a control batch were used, thus a total of eight batches of liver pâté were prepared: CON, BHT, TEA (TEA_50_, TEA_200_ and TEA_1000_) and GRA (GRA_50_, GRA_200_ and GRA_1000_). Pâté samples were analyzed following 0, 4, 8 and 24 weeks of storage. Color parameters were affected by storage period and level of antioxidant extract. Samples with TEA_200_ and GRA_1000_ levels of extracts showed lower total color difference between 0 and 24 weeks. At the end of storage period, the lower TBARs values were obtained in samples with the highest concentration on natural extract. Overall, the evolution of volatile compounds showed an increase in those ones that arise from the lipid oxidation and samples with TEA_1000_ extract showed the lowest values.

## 1. Introduction

The prevention of lipid oxidation along the lines of processing and storage of meat products is important with regards to its quality and healthiness [1]. Nitrites have been widely used in cooked meat products alone or joined to natural antioxidants [2]. However, the potential health risks related to residual nitrite levels and the formation of harmful *N*-nitrosamines in meat products has led the meat industry to reduce the use of sodium nitrite, significantly. On the other hand, due to the fact that synthetic antioxidant may constitute a potential health hazard for consumers, interest in natural antioxidants and search of naturally occurring compounds with antioxidant activity has increased dramatically [3,4].

In the last years, many researchers have evaluated the antioxidant capacity of extracts from different plants derived foods; specifically studies with grape and tea extracts have been developed in meat patties [1,5], in cooked products [6] or even in films with positive results [7]. Concerning liver pâtés and to our knowledge, in only one study has been employed this type of extracts with levels of 1000 ppm to prove the extract efficacy [8]. This level is necessary due to chemical composition of pâtés (high amounts of fat and non-heme iron, and low content of natural antioxidants) and their manufacturing process (high temperature) this product is highly susceptible to lipid oxidation [2,9]. However, the disadvantages of the use of natural antioxidants are that they are usually more expensive than synthetic antioxidants and may impart color or taste to the product [3] so studies that investigate the dose effect in “real” (*i.e.*, food) matrix are necessary to obtain a better extract characterization. In addition, many of them are by-products of agro-industries (e.g., grape extract) and their use could represent a significant step towards an effective and economically valuable valorization of such waste [10].

The aim of this work was therefore to evaluate the effect of the addition of increasing amounts of natural antioxidants (tea (TEA) and grape seed (GRA) extracts) on the physiochemical and oxidative stability of refrigerated stored pig pâtés, and to compare the effects observed with those of a synthetic antioxidant (butylated hydroxytoluene BHT) and a control (CON).

## 2. Experimental Section

### 2.1. Grape Seed Extract (GRA) and Green Tea Extract (TEA)

The procedures for the preparation of the TEA and GRA extracts were carried out as previously described [1,10].

### 2.2. Determination of Antioxidant Activity

The antioxidant activity of these extracts was previously described by Pateiro *et al.* [8].

### 2.3. Manufacture of Liver Pâté

For this study, eight batches of liver pâté were prepared: CON (control), BHT (BHT, 200 mg/kg), TEA (tea extract; 50, 200 and 1000 mg/kg; hereafter TEA_50_, TEA_200_ and TEA_1000_, respectively) and GRA (grape seed extract; 50, 200 and 1000 mg/kg of liver pate; hereafter GRA_50_, GRA_200_ and GRA_1000_, respectively). The pâtés were prepared in the pilot plant of the Meat Technology Center of Galicia. An identical formula was used for all batches, except for the addition of the different antioxidants. The ingredients (%) were as follows: subcutaneous fat (40%), lean meat (15%), liver (18%), cold water (23%), sodium chloride (2%), and sodium caseinate (2%). Fat, meat and liver were from the Celta pig breed. First, fat and liver were chopped in to small cubes and scalded at 65 °C for 10 min. The cooked fat and liver, after being allowed to cool at room temperature, were mixed with the remaining ingredients in a Talsa bowl chopper (Talsabell, S.A., Valencia, Spain). After that, the total mass was divided in five batches of 3 kg each. Antioxidants (BHT, tea, and grape seed extract) were added in the corresponding batch (BHT, TEA and GRA, respectively) and the mass was mixed with a beater. Finally, the mixture was packed in glass containers (150 g) and cooked by immersion in a hot water bath at 80 °C for 30 min. After the meat samples were allowed to cool at room temperature, they were stored in the dark at 4 °C for 24 weeks. Batches were made in triplicate. Two units of pâté from each batch were taken at 0, 4, 8 and 24 weeks to determine the following parameters: pH, color, thiobarbituric acid reactive substances (TBARs), and fatty acid composition. The volatile compounds profile of the manufactured liver pâté was determined at the beginning and at the end of the storage period.

### 2.4. Analytical Methods

Analytical procedures (color parameters, TBARS index, fatty acid and volatile profile) were carried out as previously described by Pateiro *et al.* [8].

#### 2.4.1. Physical Analysis

The pH of samples was measured using a pH-meter (HI 99163, Hanna Instruments, Eibar, Spain) equipped with a glass probe for penetration.

A portable colorimeter (Konica Minolta CM-600d, Osaka, Japan) with the next settings machine (pulsed xenon arc lamp, 0° viewing angle geometry, standard illuminant D65 and aperture size of 8 mm) was used to measure the pâté color in the CIELAB space. Results were expressed as lightness (L*), redness (a*) and yellowness (b*).

The total color difference (ΔE) between pâtés at day 0 and week 24 of storage was calculated by the next formula [11].

$$\Delta E_{0-24}={\left[{\left(L_{24}-L_{0}\right)}^{2}+{\left(a_{24}-a_{0}\right)}^{2}+{\left(b_{24}-b_{0}\right)}^{2}\right]}^{1/2}$$

The relative content of metmyoglobin (METOX) on the surface of the samples is based on measurements of reflex attenuance of incident light at the isobestic points 572, 525, 473 and 730 nm [12].

#### 2.4.2. Lipid Oxidation

Lipid stability was evaluated using the method proposed by Vyncke [13]. Thiobarbituric acid reactive substances (TBARs) were calculated from a standard curve of malonaldehyde (MDA) produced from with 1,1-3,3 tetraethoxypropane (TEP) and expressed as mg MDA/kg sample.

#### 2.4.3. Analysis of Fatty Acid Methyl Esters

The fat was extracted using chloroform/metanol (2/1; v/v) and stored at −80 °C until analysis. Lipids were trans-esterified with a solution of boron trifluoride (14%) in methanol. For total fatty acids analysis, 50 mg of the extracted lipids were esterified to form fatty acid methyl esters (FAMEs) which were stored at −80 °C until chromatographic analysis. Separation and quantification of the FAMEs were carried out using a gas chromatograph (GC-Agilent 6890N; Agilent Technologies Spain, S.L., Madrid, Spain) equipped with a flame ionization detector and an automatic sample injector HP 7683, and using a Supelco SPTM-2560 fused silica capillary column (100 m, 0.25 mm i.d., 0*.*2 μm film thickness, Sigma-Aldrich, Spain). The chromatographic conditions were as follows: the initial column temperature (120 °C) was maintained for 5 min, then programmed to increase at a rate of 5 °C/min up to 200 °C maintaining this temperature for 2 min, then at 1 °C/min up to 230 °C maintaining this temperature for 3 min, and then increasing again at 2 °C/min up to a final temperature of 235 °C which is then held for 10 min. The injector and detector were maintained at 260 and 280 °C, respectively. Helium was used as carrier gas at a constant flow-rate of 1.1 mL/min, with the column head pressure set at 37.73 psi. The split ratio was 1:50 and 1 μL of solution was injected. Nonanoic acid methyl ester (C9:0 ME) at 0.3 mg/mL was used as internal standard and added to the samples prior to injection. Individual FAMEs were identified by comparing their retention times with those of authenticated standards (Supelco 37 component FAME Mix).

Data regarding FAME composition were expressed as percentage of total area of FAMEs. The proportion of polyunsaturated (PUFA), monounsaturated (MUFA), and saturated (SFA) fatty acid contents and the ratios PUFA/SFA, *n*-6/*n*-3 and nutritional value (NV) = ∑(C14:0 + C16:0)/∑(C18:1 + C18:2*n*6c) were calculated.

#### 2.4.4. Analysis of Volatile Compounds

The volatile compounds profile was studied at the beginning and the end of the refrigerated storage. The extraction of the volatile compounds was performed using solid-phase microextraction (SPME). For headspace SPME (HS-SPME) extraction, 2 g of each ground sample were weighed in a 40 mL vial and screw-capped with a laminated Teflon-rubber disk. The fiber, previously conditioned by heating in a gas chromatograph injection port at 270 °C for 60 min, was inserted into the sample vial through the septum and then exposed to headspace. The extractions were carried out in an oven at 35 °C for 30 min, after equilibration of the samples for 15 min at the extraction temperature, ensuring a homogeneous temperature for sample and headspace. Once sampling was finished, the fiber was withdrawn into the needle and transferred to the injection port of the gas chromatograph−mass spectrometer (GC−MS) system.

A gas chromatograph 6890N (Agilent Technologies, Santa Clara, CA, USA) equipped with a mass selective detector 5973N (Agilent Technologies) was used with a DB-624 capillary column of 30 m × 0.25 mm i.d., 1.4 μm film thickness (J & W Scientific, Folsom, CA, USA). The SPME fiber was desorbed and maintained in the injection port at 260 °C during 5 min. The sample was injected in splitless mode. Helium was used as a carrier gas with a linear velocity of 40 cm/s. The temperature program was isothermal for 10 min at 40 °C, raised to 200 °C at a rate of 5 °C/min, and then raised to 250 °C at a rate of 20 °C/min and held for 5 min. Injector and detector temperatures were both set at 260 °C. The mass spectra were obtained using a mass selective detector at 70 eV, with a multiplier voltage of 1953 V and collected data at a rate of 6.34 scans/s over a mass range of m/z 40–300. Compounds were identified comparing their mass spectra with those contained in the NIST05 (National Institute of Standards and Technology, Gaithersburg, MD, USA) library and/or by calculation of their retention index relative to a series of standard alkanes (C5–C14) for calculating the Kovats indexes and matching them with data reported in literature. Results for each volatile compound were the mean value of three replicates and they were expressed as AU (area units) ×10^6^.

### 2.5. Statistical Analysis

For the statistical analysis of the results, one way analysis of variance (ANOVA) using SPSS package (SPSS 19.0, Chicago, IL, USA) was performed for all variables considered in the study. Least-squares means were compared among treatments using the Duncan’s post hoc test for a significance level *p* < 0.05. Correlations between variables were determined using correlation analyses using Pearson’s correlation coefficient (r) with the above mentioned statistical software package.

## 3. Results and Discussion

### 3.1. Antioxidant Activity of the Extracts

GRA and TEA extracts showed a high polyphenol content (373.0 *vs.* 390.0 mg GAE/g extract, respectively), mainly flavonoids and flavan-3-ols, which antioxidant activity has been demonstrated [14,15]. The major compounds found in TEA extracts were catechin, epicatechin, cinnamic acids and sugar-linked flavonols [16], while GRA extracts contained benzoic acids, monomer flavan-3-ols and oligomeric procyanidins [15]. The higher activity found in GRA extracts could be associated to its resveratrol content [17].

TEAC, DPPH and β-carotene were used to assess *in vitro* antioxidant activity of the natural extracts. These methods were directly related to polyphenol contents [8]. In the case of TEAC, the aforementioned extracts displayed values of 2.93 and 4.06 g Trolox/g extract for GRA and TEAextracts, respectively.

The scavenging activity found on DPPH radical showed the higher antioxidant power of BHT standard, followed by GRA and TEA extracts (1.80 and 2.18 g equivalent BHT/g extract, respectively). The EC_50_ values obtained showed values of 0.12 and 0.16 g extract/L for TEA and GRA extracts, respectively.

β-carotene bleaching assay of the natural extracts showed that GRA was the most active (1.28 g equivalent BHT/g extract), while TEA displayed values of 0.69 g equivalent BHT/g extract. The EC_50_ values obtained displayed rather similar activity values for both extracts (less than 0.10 g extract/L).

### 3.2. Color Properties of Pig Liver Pâtés during Storage Time

Except for week 0, no significant differences (*p* > 0.05) were detected for pH values among batches during refrigerated storage (Table 1). Samples with antioxidants displayed pH values lower than the CON batch. This fact could be due to the acidity of the antioxidant extracts (pH of 4.29 and 5.74 for GRA and TEA extracts, respectively), conferring a lower pH on the samples containing antioxidants in their formulation. Regarding the dose used of each antioxidant, a reduction in pH was observed with increasing concentration (Table 1). Colour parameters were significantly (*p* < 0.05) influenced by the period of storage. Significant differences were found for L* values in the samples treated with BHT and with low concentrations of natural antioxidants (*p* < 0.05) within each group. However, no significant differences (*p* > 0.05) were observed for L* values among the antioxidant levels.

In our study, the lowest values for L* were found in the samples treated with 1000 mg/kg, obtaining values of 62.34 and 64.40 for samples treated with TEA and GRA extracts, respectively, compared to values of 70.56 and 70.03 observed in samples treated with 50 mg/kg of TEA and GRA extracts, respectively. These findings are in agreement with other authors who observed lower L* values in pâté samples with increasing amounts of natural antioxidant [18]. The effect was compared with that of BHT at the same dose (200 mg/kg), TEA and GRA extracts showed lower L* values than BHT (68.43 and 69.81 *vs.* 71.39 for TEA, GRA and BHT, respectively). Finally, similar values were found between CON and samples treated with the lowest concentration of natural antioxidants (50 mg/kg) (71.39 *vs.* 70.56 and 70.03 for TEA and GRA, respectively).

Regarding redness, its development was different depending on the batch. Many differences were observed among the antioxidant used, which could be due to the colour that the addition of the antioxidant may give to the final product. At the beginning of the period of storage, the values ranged between 1.59 and 4.63, with the highest values obtained for CON and BHT and the lowest obtained for TEA_50_ and GRA_50_ (4.63 and 4.61 *vs.* 3.66 and 4.12 for CON, BHT, TEA_50_ and GRA_50_ batches, respectively). At the end of the period of storage, samples treated with BHT showed the highest values, followed by CON, TEA and GRA at the lowest concentration (4.73 and 4.58 *vs.* 3.63 and 3.72 for BHT, CON, TEA_50_ and GRA_50_ batches, respectively). For the dose effect, as happened with the L* values, the higher the amounts of the natural antioxidants the lower the a* values of the pate samples. The trend observed for samples treated with TEA extract were similar to that observed for samples treated with GRA.

Concerning yellowness (b*), the obtained values decreased during the period of storage, although only significant (*p* < 0.05) differences were found in BHT, TEA_50_ and TEA_1000_ batches. Unlike other studies, the b* values were lower in pâtés to which the natural antioxidants were added than in CON and BHT samples [19]. As with L* and a* values, the greater the amount of natural antioxidant added to the pâté, the lower the b* values of the sample. TEA_1000_ and GRA_1000_ samples had the lowest values at the beginning and at the end of the storage period. At the end of the period of storage, TEA_50_ and GRA_50_ treatments showed similar b* values to those of the CON and BHT batches, with values of 17.77 and 18.11 *vs.* 18.35 and 18.71 for TEA_50_, GRA_50_, CON and BHT batches, respectively. Values obtained for the total color difference (ΔE) between pâtés at day 0 and each sampling point showed that samples treated with natural antioxidants had the lowest color differences during storage (Table 1). In fact, the changes observed in the color parameters were less in TEA_200_, GRA_200_ and GRA_1000_ batches. These color differences can be considered noticeable when ΔE values are higher than 2 [20]. The color changes that occurred during the storage period could be associated with oxidation processes and are contrary to what other authors observed in porcine liver pâtés [19].

**Table 1:** Effect of antioxidants on physical properties of Celta pig liver pâtés (*n* = 3) during refrigerated storage.

		CON	BHT	TEA			GRA			*p*-Value	SEM
				50	200	1000	50	200	1000		
***pH***	0	6.34 ^a,5^	6.27 ^a,4^	6.29 ^4^	6.21 ^2,3^	6.18 ^a,b,1,2^	6.23 ^3^	6.20 ^a,b,2,3^	6.16 ^b,1^	0.000	0.02
	4	6.30 ^b,c,2^	6.16 ^b,1,2^	6.00 ^1^	6.14 ^1,2^	6.14 ^b,1,2^	6.17 ^1,2^	6.17 ^b,1,2^	6.13 ^c,1,2^	0.207	0.03
	8	6.27 ^c^	6.18 ^b^	6.13	6.06	6.07 ^c^	6.22	6.19 ^b^	6.12 ^c^	0.069	0.02
	24	6.34 ^a,b^	6.25 ^a^	6.15	6.22	6.21 ^a^	6.25	6.23 ^a^	6.20 ^a^	0.071	0.02
	*p-value*	0.025	0.009	0.306	0.296	0.003	0.217	0.031	0.001		
	*SEM*	0.01	0.02	0.05	0.03	0.02	0.01	0.01	0.01		
L*	0	67.64 ^c,4^	74.50 ^a,7^	76.06 ^a,8^	66.88 ^3^	58.01 ^b,1^	74.38 ^a,6^	69.71 ^5^	65.21 ^2^	0.000	1.45
	4	69.34 ^b,c,4^	68.16 ^b,3,4^	67.49 ^b,3,4^	65.81 ^2,3^	61.62 ^a,1^	70.10 ^b,4^	67.14 ^2–4^	63.87 ^1,2^	0.004	0.73
	8	70.87 ^a,b,2^	71.67 ^a,2^	71.77 ^b,2^	68.81 ^2^	61.55 ^a,1^	72.17 ^a,b,2^	70.29 ^2^	64.51 ^1^	0.001	0.98
	24	71.39 ^a,2^	71.39 ^a,2^	70.56 ^b,2^	68.43 ^2^	62.34 ^a,1^	70.03 ^b,2^	69.81 ^2^	64.40 ^1^	0.000	0.85
	*p-value*	0.014	0.024	0.022	0.334	0.010	0.065	0.306	0.397		
	*SEM*	0.58	0.90	1.23	0.62	0.66	0.75	0.62	0.26		
a*	0	4.63 ^a,7^	4.61 ^a,b,7^	3.66 ^a,5^	2.43 ^3^	1.85 ^a,2^	4.12 ^6^	3.17 ^4^	1.59 ^1^	0.000	0.29
	4	4.43 ^a,4^	4.36 ^a,b,4^	3.37 ^a,b,3^	2.47 ^2^	1.47 ^b,1^	3.38 ^3^	3.25 ^3^	1.21 ^1^	0.000	0.29
	8	3.96 ^b,4,5^	4.09 ^b,5^	3.24 ^b,3^	1.93 ^2^	1.12 ^c,1^	3.67 ^4^	3.00 ^3^	1.04 ^1^	0.000	0.30
	24	4.58 ^a,4,5^	4.73 ^a,5^	3.63 ^a,b,3,4^	2.46 ^2^	1.13 ^c,1^	3.72 ^3,4^	3.38 ^2,3^	1.20 ^1^	0.000	0.34
	*p-value*	0.024	0.099	0.116	0.243	0.011	0.359	0.686	0.307		
	*SEM*	0.11	0.11	0.08	0.11	0.12	0.14	0.10	0.10		
b*	0	19.45 ^a,7^	20.18 ^a,8^	18.79 ^a,6^	15.58 ^3^	11.96 ^a,1^	18.53 ^5^	17.06 ^4^	13.51 ^2^	0.000	0.71
	4	18.78 ^a,b,5^	19.22 ^b,5^	17.44 ^b,4^	15.86 ^3^	11.35 ^a,b,1^	17.57 ^4^	17.49 ^4^	12.99 ^2^	0.000	0.68
	8	18.97 ^a,b,5^	19.48 ^a,b,5^	17.73 ^b,4^	15.25 ^3^	10.26 ^c,1^	18.51 ^4,5^	17.79 ^4^	12.93 ^2^	0.000	0.80
	24	18.35 ^b,5^	18.71 ^b,5^	17.77 ^b,4,5^	15.63 ^3^	10.45 ^b,c,1^	18.11 ^4,5^	17.21 ^4^	12.86 ^2^	0.000	0.73
	*p-value*	0.110	0.033	0.023	0.464	0.027	0.363	0.263	0.190		
	*SEM*	0.17	0.22	0.21	0.13	0.28	0.21	0.14	0.12		
**Δ*E***	0-4	1.89 ^1,2^	6.42 ^3,4^	8.70 ^4^	1.14 ^1^	3.68 ^1–3^	4.52 ^2,3^	2.62 ^1,2^	1.50 ^1,2^	0.005	0.68
	0-8	3.36	2.97	4.77	2.34	4.01	2.37	1.21	1.07	0.252	0.39
	0-24	3.91 ^1,3^	3.47 ^2,3^	5.59 ^4^	1.87 ^1^	4.65 ^3,4^	4.42 ^3,4^	2.06 ^1,2^	1.24 ^1^	0.001	0.40
	*p-value*	0.122	0.148	0.173	0.759	0.559	0.313	0.136	0.860		
	*SEM*	0.44	0.80	0.96	0.54	0.32	0.60	0.30	0.26		

^a**–**c^ Mean values in the same column (same antioxidant in different weeks) with different letter indicating significant differences; ^1**–**8^ Mean values in the same row (different antioxidant in the same week) with different number indicating significant differences; SEM: standard error of mean; Batches: CON: control; BHT: tert-butyl-4-hydroxytoluene; TEA: tea and GRA: grape seed extracts.

On the contrary, TEA_50_ and GRA_50_ batches displayed higher ΔE_0–24_ than the CON batch. In this case, color changes could be associated with some compositional or physical changes that happened during period of storage which are not directly related to oxidative processes. In this regard, Estévez *et al.* [19] also observed this behavior in porcine liver pâtés prepared with rosemary and sage extracts. However, the color change (ΔE_0–24_) of pâtés treated with TEA_1000_ and GRA_1000_ extracts were lower than those observed by the aforementioned authors in samples prepared with the same concentration (0.1%) of rosemary and sage extracts [19]. Finally, the values found for ΔE_0–24_ in BHT batches were lower than those found in other liver pâtés (3.47 *vs.* 5.45) [19], while the values observed for CON batches were higher (3.91 *vs.* 3.38). Among samples, no significant differences (*p* > 0.05) were found during the storage period; the lowest ΔE_0–24_ values were for samples with GRA extract followed by TEA and BHT batches.

Within the dose effect, as happened with L* and a* values in GRA batches, the greater the amount of natural antioxidant added to the pâté, the lower was ΔE value of the sample. This behavior was also observed in frankfurters treated with increasing levels of rosemary essential oil [21]. On the contrary, the results obtained for TEA batches showed an increase in ΔE_0–24_ for the highest concentration.

### 3.3. Oxidative Stability of Pig Liver Pâtés during Storage Time

**Figure 1:**
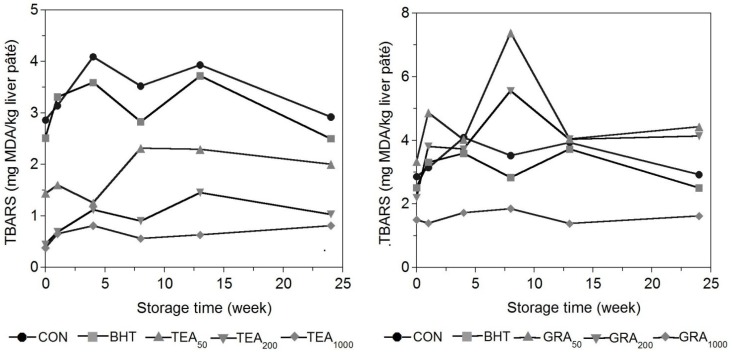
Evolution of TBARs during refrigerated storage of porcine liver pâté with added BHT and natural antioxidants.

The oxidative stability of pig liver pâtés was measured based on the TBARs index (Figure 1), which is frequently used as a marker of lipid oxidation [22]. The results obtained show that TBARs were unstable during processing, although the overall trend was an increase during the storage period. This rise was expected to occur during refrigerated storage of pâtés as a result of the onset of oxidative reactions following cooking [23]. The conditions used for the thermal treatment (80 °C for 30 min) (see the experimental section) are commonly used in the manufacture of liver pâtés [8,9,19,24].

Different values were observed among treatments during the period of storage. At day 0, TBARs values of all samples ranged from 0.4 to 3.3 mg malondialdehyde (MDA)/kg. At the end of the storage period, the lower TBARs values were obtained in samples with TEA: 2.00, 1.03 and 0.81 mg MDA/kg *vs.* 2.92 mg MDA/kg for TEA_50_, TEA_200_, TEA_1000_ and CON batches, respectively. This behavior was also observed by other authors in goat meat sausages treated with 0.025% and 0.05% of rosemary extract [25]. These results are in agreement with previously published studies reporting the greater effectiveness of the natural antioxidants compared to the synthetic antioxidant supports the possibility of using these extracts in place of commercially used synthetic antioxidants such as BHT [9,26].

On the other hand, results revealed that TBARs values were affected by the period of storage within each batch (*p* < 0.05), increasing at the beginning of storage period and decreasing after reaching halfway through the storage period (at week eight). This trend could be due to oxidative reactions beginning during processing and are directly correlated with the protective effects of the antioxidant and indicate that lipid oxidation begins during the processing of ingredients (scalding of fat and liver) and before addition of antioxidants. Similar TBARs graphs have been obtained for other meat products that have undergone thermal treatments, because the exposure to high temperatures (>70 °C) is a strong promoter of lipid oxidation [27]. Some authors suggested that the decrease in the TBARs index occurs when the reaction rate of the carbonyls in proteins becomes higher than the rate of TBARs formation [28].

### 3.4. Relationship between TBARs and METOX Production and the Concentration of the TEA and GRA Extracts

**Figure 2:**
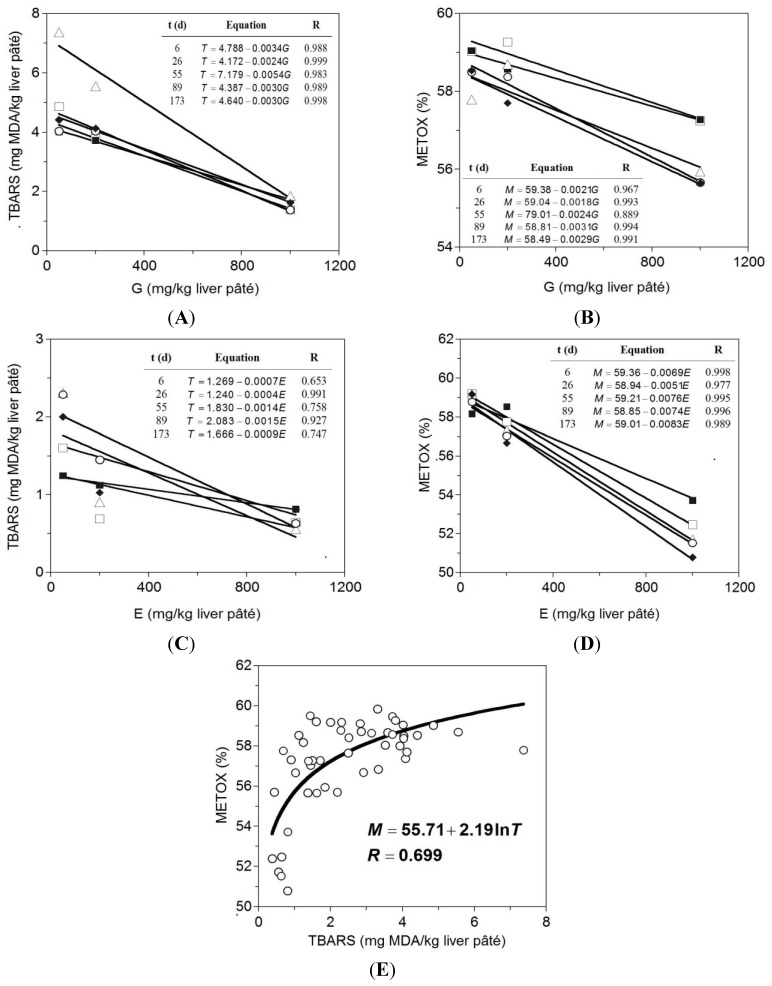
Linear relationships among TBARs and METOX production and extracts concentrations (tea: *E* and grape: *G*) at different storage times: 6 (□), 26 (■), 55 (∆), 89 (○) and 173 days (♦), and non-linear relationship among TBARs and METOX production data. (**A**) TBARs for grape extracts application; (**B**) METOX for grape extracts application; (**C**) TBARs for tea extracts application; (**D**) METOX for tea extracts application and (**E**) TBARS *vs.* METOX production.

METOX values are related to oxidative stability, which is a marker of food deterioration [29]. METOX is also related to redness values [30]. In general, the higher the amount of natural antioxidant added to the pâté, the lower was TBARs and METOX values of the sample (Figure 2). The results obtained of TBARs evolution showed the ability of these natural antioxidants to reduce the oxidative deterioration of lipids. In some cases, TEA extracts improved the results obtained by BHT (even at a lower concentration). Although, with a low number of freedom degrees, the correlation of TBARs and METOX trends *vs.* extracts concentration was mainly linear (Figure 2). The degree of determination was high in almost all responses (*R*^2^ > 0.94) less for the case of TBARs production influenced by TEA extracts. Several works showed similar capacity of natural extracts to prevent lipid oxidation on meat products [27,31]. As in the present study, in many reports the improving effects of extracts were concentration-dependent [6,32], descending the production of TBARs at increasing levels of antioxidants. Nevertheless, our results were not in agreement with those found by other authors who observed an increment of TBARs and METOX in minced beef patties with higher concentration of tea catechins [5]. The storage time also revealed to be a factor that enhanced the value of the slope from first order equations (Figure 2) and therefore the suitability to reduce the oxidation process in meat products. However, no significant (*p* > 0.05) effect of the storage time on the slope of the TBARs *vs.* grape extracts equation was observed. Finally, the non-linear relationship between data of TBARs (*T*) and METOX (*M*) were described by means of a logarithmic equation: *M* = 55.71 + 2.19 ln *T*.

Although the coefficient of determination was poor (*R*^2^ = 0.488), the consistency of the equation was good (*p* < 0.05) and the experimental data trend was clearly non-linear. This result is not in concordance with the affirmation, extensively reported in the literature, of linearity correlation among both variables [33,34]. However, similar low correlations and logarithmic relationships were also established for the variables antioxidant vitamins and TBARs in fresh muscle treated under different experimental conditions [35].

### 3.5. Fatty Acid Composition of Pig Liver Pâtés during Storage Time

The fatty acid composition of pig liver pâtés and the most important nutritional indexes are shown in Table 2 and Table 3, respectively. The FA profile showed that MUFA were the predominant FAs, with values ranging from 50% to 60% of total methyl esters. This was followed by SFAs with values between 32.39% and 38.26% and then PUFAs with values in all cases less than 13%, following in importance.

Estévez *et al.* [4,9] also reported similar results for MUFA in porcine liver pâtés, and lower percentages (<48%) when they used white pigs for the manufacture of liver pâtés. Lower percentages were also displayed in other Spanish porcine liver pâtés [36], and when this product was manufactured using meat and liver from foals [24].

Regarding MUFA, oleic acid was the most abundant, followed by palmitoleic acid in agreement with other authors who found that oleic acid was the predominant FA in liver pâtés [4,24]. Little variations were observed for this FA based on the period of storage and among batches. Except for BHT, statistical analysis did not show significant (*p* > 0.05) differences for oleic acid concentrations during the period of storage. Among batches only significant differences (*p* < 0.05) were found at weeks 0 and 4 of the refrigerated storage. At the beginning of the period of storage, the highest values were observed in TEA_1000_ batch, with a mean value of 52.53%; while at week 24, the TEA_200_ batch had the highest values, with a mean value of 57.23%. During period of storage, CON batches had the lowest values for this FA. A similar pattern was observed for palmitoleic acid, although, in this case, BHT batch had the highest values for this FA.

**Table 2:** Effect of antioxidants on fatty acid profile of Celta pig liver pâtés (*n* = 3) during refrigerated storage.

		CON	BHT	TEA			GRA			*p*-Value	SEM
				50	200	1000	50	200	1000		
	0	20.46 ^b,1^	20.43 ^c,1^	20.70 ^b,c,5^	20.45 ^1^	20.77 ^a,b,6^	20.50 ^2^	20.58 ^3^	20.62 ^4^	0.000	0.03
***Palmitic Acid***	4	20.33 ^b,2^	20.28 ^c,2^	20.14 ^c,2^	20.22 ^2^	19.10 ^b,1^	19.85 ^2^	19.76 ^2^	19.90 ^2^	0.013	0.11
	8	23.23 ^a^	23.59 ^a^	22.89 ^a^	22.33	22.61 ^a^	22.26	22.34	22.67	0.176	0.14
***C16:0***	24	21.84 ^a,b^	21.77 ^b^	21.83 ^a,b^	18.20	20.94 ^a,b^	21.41	21.79	21.25	0.465	0.43
	*p-value*	0.039	0.002	0.026	0.225	0.042	0.103	0.228	0.068		
	*SEM*	0.48	0.51	0.43	0.70	0.51	0.40	0.48	0.43		
	0	2.45 ^3^	2.58 ^b,c,5^	2.51 ^4^	2.38 ^1^	2.54 ^4^	2.41 ^1,2^	2.39 ^1^	2.44 ^2,3^	0.000	0.02
***Palmitoleic Acid***	4	2.36 ^1^	2.51 ^c,2^	2.49 ^2^	2.41 ^1,2^	2.36 ^1^	2.35 ^1^	2.32 ^1^	2.35 ^1^	0.042	0.02
	8	2.40	2.72 ^a,b^	2.52	2.49	2.53	2.44	2.46	2.57	0.396	0.03
***C16:1***	24	2.40	2.75 ^a^	2.35	1.97	2.56	2.41	2.54	2.55	0.527	0.08
	*p-value*	0.842	0.025	0.692	0.657	0.373	0.164	0.338	0.121		
	*SEM*	0.03	0.04	0.05	0.14	0.04	0.01	0.04	0.04		
	0	12.15 ^2^	12.12 ^a,2^	12.24 ^3^	12.38 ^4^	11.42 ^1^	12.45 ^5^	12.36 ^4^	12.25 ^3^	0.000	0.08
***Stearic Acid***	4	12.73	12.15 ^a^	12.14	12.23	12.45	12.18	12.07	12.04	0.496	0.08
	8	13.43	12.42 ^a^	11.97	12.16	12.13	12.68	12.43	12.25	0.528	0.17
***C18:0***	24	12.73 ^3^	11.42 ^b,1,2^	12.05 ^2,3^	10.73 ^1^	11.40 ^1,2^	12.46 ^2,3^	12.28 ^2,3^	11.81 ^1–3^	0.029	0.18
	*p-value*	0.557	0.020	0.695	0.083	0.077	0.352	0.916	0.067		
	*SEM*	0.28	0.15	0.07	0.29	0.19	0.09	0.16	0.08		
	0	50.30 ^1^	50.60 ^a,4^	50.62 ^5^	50.66 ^5^	52.53 ^7^	50.80 ^6^	50.48 ^3^	50.45 ^2^	0.000	0.18
***Oleic Acid***	4	49.75 ^1^	50.86 ^a,1,2^	50.91 ^1,2^	50.78 ^1,2^	50.51 ^1,2^	51.80 ^2,3^	52.35 ^3^	51.55 ^2,3^	0.020	0.22
	8	47.56	46.52 ^b^	48.83	49.04	48.44	48.61	49.58	49.05	0.151	0.29
***C18:1n9***	24	49.49	50.70 ^a^	50.05	57.23	50.86	50.19	49.59	50.72	0.412	0.89
	*p-value*	0.254	0.005	0.212	0.241	0.103	0.108	0.500	0.184		
	*SEM*	0.60	0.76	0.45	1.60	0.68	0.58	0.75	0.49		
	0	10.18 ^a,5^	9.70 ^b,3^	9.53 ^2^	9.78 ^4^	9.44 ^1^	9.54 ^2^	9.70 ^3^	9.54 ^2^	0.000	0.06
***Linolei Acid***	4	10.40 ^a,2^	9.70 ^b,1^	9.62 ^1^	9.75 ^1^	9.85 ^1^	9.65 ^1^	9.63 ^1^	9.84 ^1^	0.014	0.07
	8	9.12 ^c^	10.04 ^a^	9.36	9.52	9.63	10.01	8.92	9.10	0.229	0.13
***C18:2n6***	24	9.72 ^b^	9.61 ^b^	9.87	9.76	9.59	9.46	9.66	9.56	0.639	0.05
	*p-value*	0.001	0.033	0.265	0.573	0.791	0.256	0.284	0.156		
	*SEM*	0.19	0.07	0.09	0.07	0.12	0.10	0.16	0.12		
	0	0.61 ^a,3^	0.57 ^c,2^	0.57 ^2^	0.53 ^1^	0.56 ^1,2^	0.57 ^2^	0.56 ^1,2^	0.58 ^b,2,3^	0.019	0.01
***Linolenic Acid***	4	0.53 ^b^	0.55 ^c^	0.35	0.56	0.54	0.59	0.57	0.59 ^b^	0.493	0.03
	8	0.59 ^a,b,1^	0.78 ^a,3^	0.67 ^1,2^	0.70 ^2,3^	0.70 ^2,3^	0.65 ^1,2^	0.69 ^2^	0.74 ^a,2,3^	0.022	0.02
***C18:3n3***	24	0.56 ^a,b^	0.63 ^b^	0.61	0.31	0.67	0.62	0.69	0.58 ^b^	0.540	0.04
	*p-value*	0.091	0.001	0.366	0.413	0.061	0.454	0.420	0.007		
	*SEM*	0.01	0.03	0.07	0.08	0.03	0.02	0.03	0.03		
	0	1.33 ^a,3^	1.55 ^a,5^	1.33 ^b,3^	1.43 ^a,4^	0.42 ^b,1^	1.21 ^2^	1.43 ^a,4^	1.33 ^a,3^	0.000	0.09
***Arachidonic Acid***	4	1.67 ^a^	1.70 ^a^	1.66 ^a^	1.61 ^a^	1.78 ^a^	1.14	1.00 ^a^	1.26 ^a^	0.054	0.08
	8	0.25 ^b^	0.39 ^b^	0.31 ^c^	0.27 ^b^	0.32 ^b^	0.63	0.34 ^b^	0.29 ^b^	0.066	0.03
***C20:4n6***	24	0.61 ^b^	0.44 ^b^	0.53 ^c^	0.40 ^b^	0.53 ^b^	0.71	0.53 ^b^	0.66 ^a,b^	0.988	0.07
	*p-value*	0.003	0.004	0.001	0.014	0.018	0.173	0.009	0.052		
	*SEM*	0.22	0.24	0.21	0.24	0.24	0.12	0.17	0.18		

^a–c^ Mean values in the same column (same antioxidant in different weeks) with different letter indicating significant differences; ^1–6^ Mean values in the same row (different antioxidant in the same week) with different number indicating significant differences; SEM: standard error of mean; Batches: CON: control; BHT: tert-butyl-4-hydroxytoluene; TEA: tea and GRA: grape seed extracts.

**Table 3:** Effect of antioxidants on nutritional properties of Celta pig liver pâtés (*n* = 3) during refrigerated storage.

		CON	BHT	TEA			GRA			*p*-Value	SEM
				50	200	1000	50	200	1000		
***SFA***	0	33.60 ^b,3^	33.55 ^b,c,2^	33.91 ^b,5^	33.72 ^4^	33.07 ^b,1^	33.92 ^a,b,5^	33.92 ^5^	33.92 ^b,5^	0.000	0.07
	4	33.92 ^b,4^	33.32 ^c,3,4^	33.25 ^b,2–4^	33.37 ^3,4^	32.39 ^b,1^	32.88 ^b,1–3^	32.63 ^1,2^	32.88 ^b,1–3^	0.010	0.13
	8	38.26 ^a^	37.74 ^a^	36.50 ^a^	36.00	36.38 ^a^	36.26 ^a^	36.27	36.49 ^a^	0.375	0.27
	24	35.85 ^a,b^	34.53 ^b^	35.14 ^a,b^	29.59	33.87 ^b^	35.16 ,^ab^	35.64	34.39 ^b^	0.349	0.66
	*p-value*	0.082	0.001	0.058	0.225	0.033	0.058	0.312	0.032		
	*SEM*	0.79	0.68	0.52	1.10	0.61	0.53	0.73	0.53		
***MUFA***	0	53.85 ^1^	54.22 ^a,5^	54.25 ^a,b,5^	54.12 ^4^	56.16 ^7^	54.32 ^a,6^	53.96 ^2^	54.07 ^3^	0.000	0.18
	4	53.11 ^1^	54.36 ^a,1–3^	54.51 ^a,1–3^	54.28 ^1,2^	53.92 ^1,2^	55.28 ^a,2,3^	55.77 ^3^	55.00 ^2,3^	0.033	0.23
	8	51.16	50.40 ^b^	52.56 ^b^	52.83	52.20	52.08 ^b^	53.21	52.83	0.265	0.30
	24	52.87	54.39 ^a^	53.44 ^a,b^	59.74	54.62	53.58 ^a,b^	53.17	54.35	0.331	0.72
	*p-value*	0.362	0.001	0.129	0.234	0.094	0.054	0.550	0.137		
	*SEM*	0.52	0.65	0.34	1.28	0.62	0.49	0.65	0.35		
***PUFA***	0	12.55 ^a,8^	11.87 ^a,7^	11.84 ^a,3^	12.16 ^6^	10.77 ^1^	11.76 ^2^	12.11 ^a,5^	11.92 ^a,4^	0.000	0.13
	4	12.97 ^a,3^	11.97 ^a,1–3^	12.05 ^a,1,2^	12.34 ^1–3^	12.56 ^2,3^	11.83 ^1,2^	11.60 ^a,b,1^	12.12 ^a,1,2^	0.049	0.12
	8	10.51 ^c^	11.39 ^a^	10.94 ^b^	11.15	11.27	11.66	10.52 ^c^	10.68 ^b^	0.117	0.15
	24	11.29 ^b^	10.73 ^b^	11.42 ^a,b^	10.67	11.34	11.26	11.13^b^	11.25 ^b^	0.627	0.09
	*p-value*	0.000	0.015	0.036	0.068	0.100	0.537	0.008	0.011		
	*SEM*	0.37	0.19	0.17	0.29	0.29	0.13	0.23	0.22		
***P/S***	0	0.37 ^a^	0.35 ^a^	0.35 ^a^	0.36	0.33 ^b^	0.35	0.36 ^a^	0.35 ^a,b^	0.319	0.01
	4	0.39 ^a^	0.36 ^a^	0.36 ^a^	0.37	0.39 ^a^	0.36	0.36 ^a^	0.37 ^a^	0.060	0.01
	8	0.28 ^c^	0.30 ^b^	0.30 ^b^	0.31	0.31 ^b^	0.32	0.29 ^b^	0.30 ^c^	0.313	0.01
	24	0.32 ^b^	0.31 ^b^	0.33 ^a,b^	0.36	0.34 ^b^	0.32	0.31 ^a,b^	0.33 ^b,c^	0.488	0.01
	*p-value*	0.002	0.003	0.048	0.098	0.030	0.170	0.059	0.014		
	*SEM*	0.02	0.01	0.01	0.01	0.01	0.01	0.01	0.01		
***n6/n3***	0	17.30 ^a,b,2^	18.34 ^a,b,4^	17.73 ^3^	19.43 ^a,6^	17.75 ^a,b,3^	17.32 ^2^	18.45 ^5^	16.73 ^1^	0.000	0.20
	4	22.12 ^a^	20.26 ^a^	18.59	18.82 ^a,b^	19.99 ^a^	16.91	17.99	17.46	0.504	2.60
	8	13.61 ^b,2^	11.73 ^c,1^	12.30 ^1,2^	12.00 ^c,1^	12.33 ^b,1,2^	16.40 ^3^	11.65 ^1^	11.30 ^1^	0.001	0.42
	24	17.27 ^a,b^	14.93 ^b,c^	16.81	17.18 ^b^	14.04 ^b^	15.61	14.66	16.63	0.976	0.78
	*p-value*	0.047	0.009	0.392	0.001	0.052	0.871	0.193	0.105		
	*SEM*	1.25	1.28	5.61	1.12	1.25	0.63	1.28	1.07		
***NV***	0	0.35 ^b^	0.35 ^b^	0.36 ^b^	0.35	0.35 ^b^	0.35	0.35	0.36 ^a,b^	0.972	0.01
	4	0.35 ^b,3^	0.35 ^b,2,3^	0.35 ^b,2,3^	0.35 ^2,3^	0.33 ^b,1^	0.33 ^1–3^	0.33 ^1,2^	0.34 ^b,1–3^	0.033	0.01
	8	0.43 ^a^	0.44 ^a^	0.41 ^a^	0.40	0.41 ^a^	0.40	0.40	0.41 ^a^	0.135	0.01
	24	0.38 ^a,b^	0.38 ^b^	0.38 ^a,b^	0.28	0.37 ^a,b^	0.37	0.39	0.37 ^a,b^	0.532	0.01
	*p-value*	0.044	0.003	0.064	0.245	0.049	0.144	0.254	0.058		
	*SEM*	0.01	0.02	0.01	0.02	0.01	0.01	0.01	0.01		

Results expressed as fatty acid percentage composition (percent by weight of total fatty acids); ^a–c^ Mean values in the same column (same antioxidant in different weeks) with different letter indicating significant differences; ^1–8^ Mean values in the same row (different antioxidant in the same week) with different number indicating significant differences; SEM is the standard error of the mean; Batches: CON: control; BHT: tert-butyl-4-hydroxytoluene; TEA: tea and GRA: grape seed extracts; SFA = ∑(C14:0 + C16:0 + C17:0 + C18:0); MUFA = ∑(C16:1 + C17:1 + C18:1 + C20:1); PUFA = ∑(C18:2*n*6 + C18:3*n*3 + C20:2 + C20:3*n*3 + C20:4*n*6); P/S = PUFA/SFA; NV: Nutritional value = ∑(C14:0 + C16:0)/∑(C18:1 + C18:2*n*6c).

The amount of SFA increased during the period of storage; palmitic and stearic acids were the predominant SFAs. Statistical analysis displayed significant (*p* < 0.05) differences for palmitic during period of storage in CON, BHT, TEA_50_ and TEA_1000_ batches. Regarding stearic acid, only significant differences (*p* < 0.05) were found in the BHT batch during the period of storage. These findings are in agreement with those reported by other authors who noticed similar percentages of SFAs in porcine liver pâtés [9]. The percentages of PUFAs were higher than those observed in other porcine liver pâtés also containing antioxidants [9] and lower than the percentages found in foal liver pâtés [24]. Linoleic acid was the predominant PUFA, with mean values around 10%. Only the BHT and CON batches showed significant (*p* < 0.05) differences during the period of storage. Among batches, significant (*p* < 0.05) differences were observed at the beginning of storage period (0 and 4 weeks). At the beginning of period of storage, the highest values were found in CON batch, with a mean value of 10.18%, while TEA_1000_ batch showed the lowest, with a mean value of 9.44%. Lower percentages of PUFA were found in arachidonic and linolenic acids, with values below 2% and 1%, respectively. Significant (*p* < 0.05) differences were observed for arachidonic acid, which decreased during the period of storage. Regarding linolenic acid, only significant (*p* < 0.05) differences were found among batches at the beginning and at week 8 of refrigerated storage.

Because of FAs contain double bonds they are targets for oxidative reactions therefore the amount of PUFAs measured is an indicator of the oxidative deterioration of meats [37]. As can be seen in Table 3, the oxidative degradation of PUFA mainly occurred after week 4. This behavior is in agreement with the results found by other authors [9] and could be attributed to the gradual degradation of endogenous antioxidants and the release of iron from the heme molecule [38]. In the present work, it can be observed that the addition of the antioxidants only protect the pâtés from oxidative degradation between week 4 and 8 of refrigerated storage, due to higher amount of PUFA found in treated samples compared to CON samples (10.51% *vs.*11.39%, 10.94%, 11.15%, 11.27%, 11.66%, 10.52% and 10.68% for CON, BHT, TEA_50_, TEA_200_, TEA_1000_, GRA_50_, GRA_200_ and GRA_1000_ batches, respectively).

To assess the nutritional properties, the ratios PUFA/SFA (P/S), *n*-6/*n*-3 and the nutritional value (NV) were determined (Table 3). PUFA/SFA ratio values were lower than the optimal values (0.5–0.7) recommended in the Mediterranean diet [39] and by the British Department of Health [40]. These ratio values decreased slightly during the period of storage, showing significant (*p* < 0.05) differences between CON and samples treated with BHT, TEA_50_, TEA_1000_ and GRA_1000_. At the beginning of the period of storage, pâtés that contained TEA_1000_ extract showed the lowest values, even though no significant (*p* > 0.05) differences were found among batches. Regarding *n*-6/*n*-3 ratio, significant (*p* < 0.05) differences were observed during refrigerated storage in CON and in the samples treated with BHT and TEA_200_, showing a decrease that was more pronounced in the BHT batch. All the *n*-6/*n*-3 ratios were higher than the nutritional recommendations of the British Department of Health [40] and *Food and Agricultural Organization* [41] for human diet, which should not exceed 4. Our results showed in all cases values above 11, which are higher than those found in foal liver pâtés [24] and Iberian liver pâtés [4]. Nevertheless, ratios reported in the present study are similar to those observed in other liver pâtés manufactured using white pigs [4]. Finally, the NV, which gives an estimation of product healthiness could have in the diet regarding its lipid content, showed a slight increase during the period of storage, mainly in CON and in the samples treated with BHT and TEA_1000_ presented significant (*p* < 0.05) differences during the period of storage.

### 3.6. Volatile Profile of Pig Liver Pâtés during Storage Time

The analysis of volatile compounds gives an indication of the chemical and metabolic processes that occur during the manufacturing process [42] and period of storage [8,36]. Also, these compounds provide information about the oxidative stability and the aroma characteristics of this product [36]. Thirty eight volatile compounds were identified from Celta pig liver pâtés samples, nevertheless only the evolution of the most abundant lipid-derived volatiles of liver pâté are shown in Table 4, being hexanal; octen-1,3-ol; hexan-1-ol and heptanal the most abundant.

Aldehydes are probably the most interesting lipid-derived volatile compounds because they can produce a wide range of flavors and odors. In the present study, almost half of the identified volatiles belonged to this family (hexanal, heptanal, and octanal). Statistical analysis showed significant (*p* < 0.001) differences for this chemical family among batches at the beginning of the storage period. The predominant aldehyde detected was hexanal, which increased significantly (*p* < 0.05) during the storage period. Hexanal as with the TBARs index is frequently used as marker of lipid oxidation due to its high sensitivity [22,43]. In fact, a statistical correlation was found between them (*r* = 0.65, *p* < 0.01). Furthermore, it is mainly generated as a result of the oxidative decomposition of PUFAs and has been related to rancid flavors [44]. Batches that contained antioxidants showed a decrease in the amount of lipid-derived volatile compounds isolated from the liver pâté (Table 4). This finding is in agreement with those reported by other authors in porcine liver pâtés [9]. Furthermore, the greater the amount of natural antioxidant added to the pâté, the lower was the concentration of aldehydes in the sample, which could equate to greater product protection.

Except for octanal, significant (*p* < 0.05) changes were observed at the end of the storage period. As happened at day 0, the addition of antioxidants as well as their use at higher concentrations of them decreased the amount of volatiles compounds isolated. These results indicate that the addition of BHT and natural antioxidants had a significant (*p* < 0.05) effect on the generation of the most volatiles compounds (Table 4). Furthermore, TEA and GRA extracts even improved the results obtained by BHT. These results are in agreement with previously published studies [9], which reported higher effectiveness of natural products compared to synthetic antioxidants and suggest the possibility of using these extracts as replacements for commercially used synthetic compounds. Compared to the CON batch, pâtés with TEA provided the most favorable results, with smaller amounts of heptanal, hexanal, hexan-1-ol and octen-1,3-ol. Furthermore, a concentration of 50 mg/kg of the natural antioxidants was enough to significantly improve the results obtained for CON and BHT batches: heptanal (6.13 and 6.02 *vs.* 7.60 and 6.70 × 10^6^ AU for TEA_50_ and GRA_50_ *vs.* CON and BHT, respectively) and hexanal (231.12 and 227.21 *vs.* 374.33 and 330.40 × 10^6^ AU for TEA_50_ and GRA_50_ *vs.* CON and BHT, respectively).

**Table 4:** Evolution of lipid-derived volatiles of Celta pig liver pâtés (*n* = 3) at day 0 and week 24 of refrigerated storage.

		CON	BHT	TEA			GRA				
				50	200	1000	50	200	1000	*p*-Value	SEM
**Week 0**	Hexanal	171.58 ^b^	159.38 ^c^	116.70 ^e^	49.22 ^g^	31.79 ^h^	233.34 ^a^	154.90 ^d^	62.33 ^f^	0.000	16.92
	2-heptanona	0.00 ^d^	0.94 ^c^	0.00 ^d^	0.00 ^d^	0.00 ^d^	2.80 ^a^	2.38 ^b^	0.00 ^d^	0.000	0.28
	Heptanal	9.34 ^a^	2.69 ^d^	2.56 ^e^	1.41 ^g^	1.22 ^h^	4.57 ^b^	4.18 ^c^	1.77 ^f^	0.000	0.64
	Octanal	0.00 ^d^	0.00 ^d^	0.00 ^d^	0.00 ^d^	0.00 ^d^	7.50 ^a^	6.94 ^b^	4.04 ^c^	0.000	0.81
	Hexan-1-ol	8.77 ^c^	5.06 ^d^	1.94 ^f^	0.00 ^g^	0.00 ^g^	9.03 ^b^	10.46 ^a^	2.89^e^	0.000	1.02
	Octen-1,3-ol	20.81 ^b^	17.21 ^c^	13.43 ^d^	5.94 ^f^	0.00 ^g^	0.00 ^g^	22.59 ^a^	7.44 ^e^	0.000	2.15
	Furan-2-penthyl	3.50 ^a^	0.74 ^c^	0.43 ^d^	0.00 ^e^	0.00 ^e^	0.75 ^c^	1.02 ^b^	0.75 ^c^	0.000	0.27
**Week 24**	Hexanal	374.33 ^a^	330.40 ^a,b^	231.12 ^b,c^	86.10 ^d,e^	24.91 ^e^	227.21 ^b,c^	182.72 ^c,d^	82.08 ^d,e^	0.001	31.22
	2-heptanona	2.90 ^a^	2.70 ^a^	1.04 ^a^	0.00 ^b^	0.00 ^b^	2.01 ^a^	2.02 ^a^	0.00 ^b^	0.002	0.32
	Heptanal	7.60 ^a^	6.70 ^a,b^	6.13 ^a,b^	2.86 ^b,c^	0.94 ^c^	6.02 ^a,b^	6.74 ^a,b^	3.36 ^b,c^	0.035	0.65
	Octanal	5.90	0.00	0.00	0.00	0.00	5.77	4.75	0.00	0.211	0.88
	Hexan-1-ol	9.47 ^a^	6.87 ^b^	8.97 ^c^	0.98 ^c^	0.95 ^c^	5.82 ^b^	6.83 ^b^	2.26 ^c^	0.000	0.80
	Octen-1,3-ol	47.41 ^a^	29.58 ^a,b^	14.10 ^b,c^	4.30 ^b,c^	2.31 ^b,c^	0.00 ^c^	10.47 ^b,c^	7.17 ^b,c^	0.025	4.53
	Furan-2-penthyl	0.87 ^a^	1.05 ^a^	0.96 ^a^	0.00 ^c^	0.00 ^c^	0.93 ^a^	1.03 ^a^	0.67 ^b^	0.000	0.11

Results expressed as AU ×10^6^; ^a–h^ Mean values in the same row (different batches on the same storage week) with different letter indicating significant differences; Batches: CON: control; BHT: tert-butyl-4-hydroxytoluene; TEA: tea and GRA: grape seed extracts.

## 4. Conclusions

From the obtained results, it can be concluded that the addition of the natural extracts improved the color stability during the period of storage, with grape extract giving the smallest color differences at the end of the period of storage. The addition of both extracts resulted in minor increases in the TBARs index and metmyoglobin percentage. Furthermore, the oxidative stability (measured in terms of amount of hexanal and other lipid-derived aldehydes such as heptanal, octanal) of the liver pates treated with natural antioxidants was significantly higher than in control sample.
